# Practical Methods to Permit the Analysis of Host Biomarkers in Resource-Limited Settings

**DOI:** 10.4269/ajtmh.21-1045

**Published:** 2022-04-18

**Authors:** Arjun Chandna, Melissa Richard-Greenblatt, Richard Tustin, Sue J. Lee, Kevin C. Kain, Sakib Burza, Yoel Lubell, Paul Turner

**Affiliations:** ^1^ Cambodia Oxford Medical Research Unit, Angkor Hospital for Children, Siem Reap, Cambodia;; ^2^ Centre for Tropical Medicine & Global Health, University of Oxford, Oxford, United Kingdom;; ^3^ University of Pennsylvania, Philadelphia, Pennsylvania;; ^4^ Children’s Hospital of Philadelphia, Philadelphia, Pennsylvania;; ^5^ Hospital of the University of Pennsylvania, Philadelphia;; ^6^ Mahidol-Oxford Tropical Medicine Research Unit, Faculty of Tropical Medicine, Mahidol University, Bangkok, Thailand;; ^7^ Department of Medicine, Division of Infectious Diseases, UHN-Toronto General Hospital, University of Toronto, Toronto, Ontario, Canada;; ^8^ Médecins Sans Frontières, New Delhi, India

## Abstract

Host biomarker testing can be used as an adjunct to the clinical assessment of patients with infections and might be particularly impactful in resource-constrained settings. Research on the merits of this approach at peripheral levels of low- and middle-income country health systems is limited. In part, this is due to resource-intense requirements for sample collection, processing, and storage. We evaluated the stability of 16 endothelial and immune activation biomarkers implicated in the host response to infection stored in venous plasma and dried blood spot specimens at different temperatures for 6 months. We found that –80°C storage offered no clear advantage over –20°C for plasma aliquots, and most biomarkers studied could safely be stored as dried blood spots at refrigeration temperatures (4°C) for up to 3 months. These results identify more practical methods for host biomarker testing in resource-limited environments, which could help facilitate research in rural and remote environments.

Increasingly, host biomarker testing is proposed as an adjunct to clinical assessment to support the management of patients with infections.^1^^–^^4^ Although different markers may serve different purposes (e.g., triage and risk stratification or guiding antimicrobial use),^5^^,^^6^ healthcare providers, policymakers, and researchers agree that one of the greatest opportunities for host biomarker measurements to contribute to patient care exists in rural areas of low- and middle-income countries (LMIC), where health worker and diagnostic capacity is most limited.^7^ However, much of the evidence on this topic is generated in urban settings.

In part, the paucity of research on this topic arises from the assumed requirements for accurate analyte quantification: prevailing practice dictates that venous blood must be centrifuged within hours of collection and plasma or serum stored at –80°C or below until assayed.^8^ However, collection of venous blood, timely centrifugation, and maintenance of a cold chain is not feasible at the peripheral levels of many LMIC health systems.

Dried blood spots (DBS) stored on filter paper, combined with less resource-intense storage methods, may overcome some of these barriers. DBS have been used successfully for the diagnosis of many tropical infections.^9^ However, certain biomarkers may not be stable when stored in field conditions.^10^ It is recommended to assess different field-to-laboratory workflows for specific analytes of interest before conducting research in remote settings.^11^^,^^12^

We evaluated the impact of different specimen types and storage temperatures (venous plasma: –80°C, –20°C; venous DBS: –20°C, 4°C, and 35°C) on the stability of 16 host biomarkers (angiopoietin [Ang]-1 [Ang-1], Ang-2, chitinase-3-like protein 1 [CHI3L1], C-reactive protein [CRP], C-X-C motif chemokine 10 [CXCL10; IP-10], interleukin [IL]-6, IL-8, IL-10, procalcitonin [PCT], soluble intercellular adhesion molecule 1 [sICAM-1], soluble tumor necrosis factor receptor 1 [sTNFR1], soluble triggering receptor expressed on myeloid cells 1 [sTREM-1], soluble vascular cell adhesion protein 1 [sVCAM-1], soluble vascular endothelial growth factor receptors 1 and 2 [sVEGFR-1, sFLT-1; sVEGFR-2, sFLT-2], and thrombomodulin [TM]) at 1-, 3-, and 6-month timepoints. Analytes were selected to align with those under evaluation in ongoing clinical studies.^13^^,^^14^

Deidentified discarded venous blood specimens from the Children’s Hospital of Philadelphia (CHOP) were used for analysis. The study did not meet criteria for human subjects’ research and was therefore exempt from CHOP institutional review board approval. All samples had been collected in ethylenediaminetetraacetic acid tubes and stored at room temperature for a maximum of 3 hours. Samples were processed immediately upon receipt to prepare plasma aliquots (seven aliquots per sample) and DBS specimens (20 DBS/sample). Multiple plasma aliquots and DBS specimens were prepared from the same original sample to avoid freeze–thaw cycles (Figure 1).

**Figure 1.**
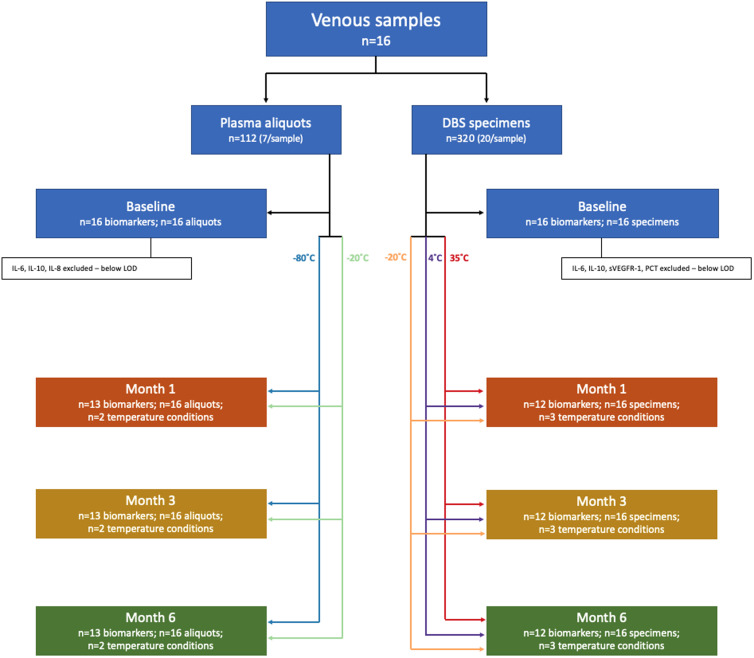
Flow diagram to illustrate study workflow. DBS = dried blood spot; LOD = limit of detection. This figure appears in color at www.ajtmh.org.

DBS specimens were prepared by spotting 50 µL of whole blood onto Whatman Protein Saver 903 Cards and left to air dry on drying racks overnight. Spots were then stored in gas-impermeable bags containing a desiccant pouch and humidity indicator card at –20°C, 4°C, and 35°C. Bags were monitored weekly. Plasma specimens were prepared by centrifuging whole blood at 1,100 to 1,300*g* for 20 minutes at ambient temperature. Aliquots were stored immediately at –20°C and –80°C.

The concentrations of host biomarkers were measured at each timepoint and storage temperature using the multiplex Magpix Luminex platform (Austin, TX) with custom-developed reagents from R&D systems (Minneapolis, MN), according to manufacturer recommendations. For DBS, specimens were extracted from the filter paper as described in Appendix 1 (see online supplementary materials for appendices) before biomarker analysis. Appropriate dilutions were performed to ensure analyte concentrations fell within the dynamic range of the Magpix assay (Appendix 1). DBS specimens and plasma aliquots were run in duplicate and mean analyte concentration calculated for analysis.

Of the 16 biomarkers of interest, IL-6, IL-8, and IL-10 were excluded for the plasma aliquots, and IL-6, IL-10, PCT, and sVEGFR-1 were excluded for the DBS specimens because too few samples had detectable levels at baseline (Figure 1). For the remaining 14 biomarkers, fractional change from baseline concentration was calculated at each timepoint for each biomarker-specimen type. For each sample, slopes were calculated to indicate change in biomarker concentration over time. Samples with baseline values below the lower limit of quantification (LLOQ) were excluded for that particular biomarker-specimen-temperature type (*N* = 104 of 996; 10.4%) (Appendix 2). Values below the LLOQ at 1, 3, or 6 months were set to the LLOQ (*N* = 39 of 2,664; 1.5%).

Although the assay manufacturer reports 30% interassay variation for analytes,^15^ we conservatively considered the time at which any change > 20% from baseline occurred as evidence of potentially important longitudinal instability. Median slopes for each biomarker specimen temperature type were compared against plasma stored at –80°C using the Wilcoxon matched-pairs signed-rank test.

Of the 13 biomarkers assessed in plasma, only sICAM-1 was unstable, with median fractional change from baseline exceeding 20% between 3 and 6 months, at both –80°C and –20°C. For the DBS specimens, 10 of 12 biomarkers (Angpt-2, CHI3L1, CXCL10, IL-8, sICAM-1, sTNFR1, sTREM-1, sVCAM-1, sVEGFR-2, TM) were stable for 1 month at all temperatures, and two biomarkers (CHI3L1, sTREM-1) demonstrated stability to 6 months. At storage temperatures ≤ 4°C, all biomarkers in DBS were stable for 1 month, with 5 (CHI3L1, sTNFR1, sTREM-1, sVCAM-1, TM) stable to 6 months. When DBS were stored at –20°C, two-thirds of the biomarkers (Angpt-1, Angpt-2, CHI3L1, CRP, sTNFR1, sTREM-1, sVCAM-1, TM) were stable for 6 months (Figure 2). Recommendations based on these results are summarized in Table 1. Each individual sample’s fractional (fitted and absolute) and actual change from baseline at each timepoint is presented in Appendix 3, by biomarker and specimen type.

**Figure 2.**
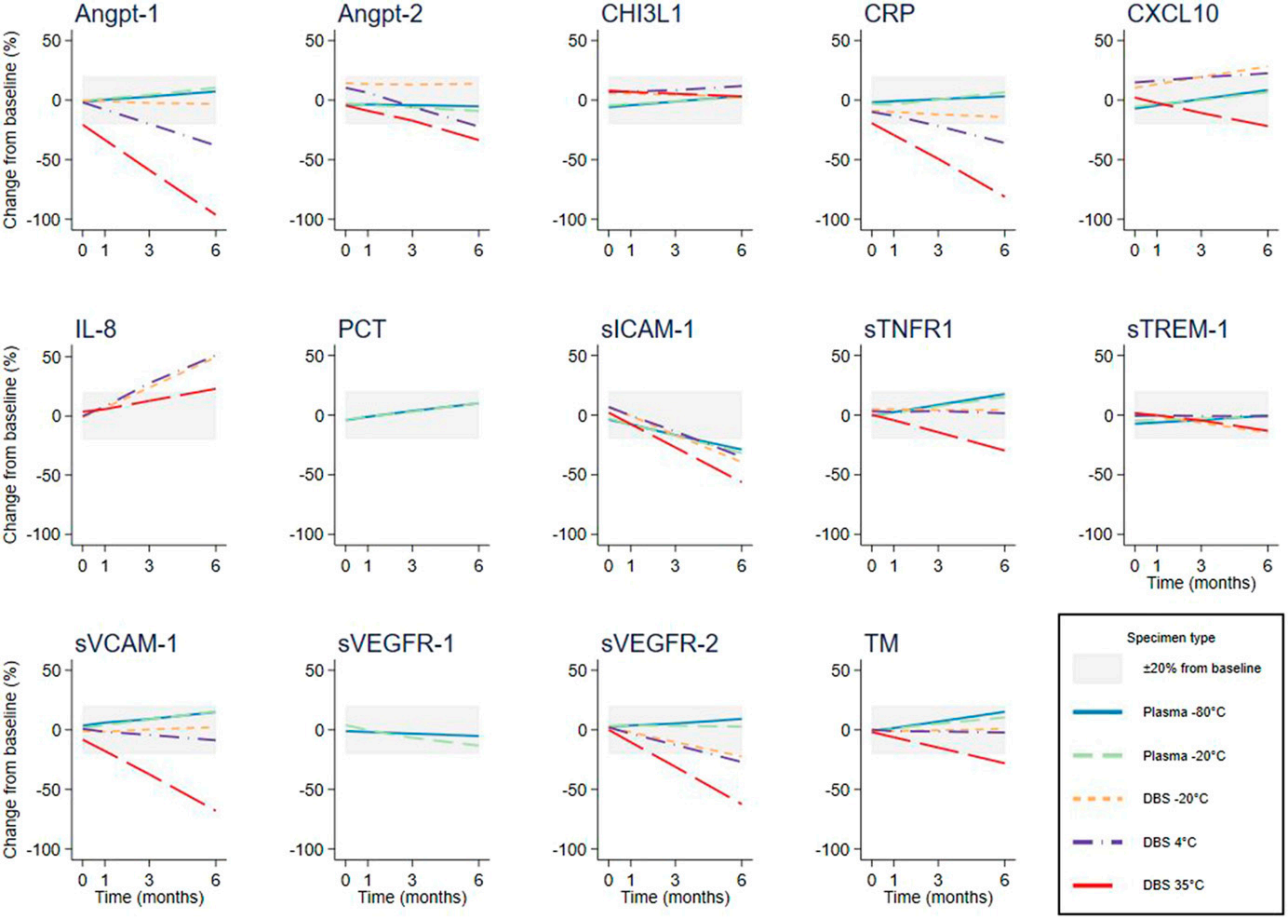
Rate of fractional change in concentration for 14 host biomarkers assayed from venous plasma and dried blood spot (DBS) specimens stored at different temperatures over 6 months. Lines indicate median fractional change from baseline concentration; solid blue = plasma –80°C; dashed green = plasma –20°C; short-dash orange = DBS –20°C; dash-dot purple = DBS 4°C; long-dash red = DBS 35°C. Gray rectangles indicate ±20% change from baseline concentration. IL-8 was only tested in DBS, and PCT and sVEGFR-1 only tested in plasma. Angpt = angiopoietin; CHI3L1 = chitinase-3-like protein 1; CRP = C-reactive protein; CXCL10 = C-X-C motif chemokine 10; IL = interleukin; PCT = procalcitonin; sICAM-1 = soluble intercellular adhesion molecule 1; sTNFR1 = soluble tumor necrosis factor receptor 1; sTREM-1 = soluble triggering receptor expressed on myeloid cells 1; sVCAM-1 = soluble vascular cell adhesion protein 1; sVEGFR-1 and -2 = soluble vascular endothelial growth factor receptors 1 and 2; TM = thrombomodulin. This figure appears in color at www.ajtmh.org.

**Table 1:** Recommended duration of storage for 14 host biomarkers under study in months

Analyte	Plasma		Dried blood spot		
	–80°C	–20°C	–20°C	4°C	35°C
CHI3L1	6	6	6	6	6
sTREM-1	6	6	6	6	6
sTNFR1	6	6	6	6	3
TM	6	6	6	6	3
sVCAM-1	6	6	6	6	1
Angpt-2	6	6	6	3	3
Angpt-1	6	6	6	3	–*
CRP	6	6	6	1	–*
CXCL10	6	6	3	3	3
sVEGFR-2	6	6	3	3	1
sICAM-1	3	3	3	3	1
IL-8	NA	NA	1	1	1†
PCT	6	6	NA	NA	NA
sVEGFR-1	6	6	NA	NA	NA

Angpt = angiopoietin; CHI3L1 = chitinase-3-like protein 1; CRP = C-reactive protein; CXCL10 = C-X-C motif chemokine 10; IL = interleukin; NA = not available; PCT = procalcitonin; sICAM-1 = soluble intercellular adhesion molecule 1; sTNFR1 = soluble tumor necrosis factor receptor 1; sTREM-1 = soluble triggering receptor expressed on myeloid cells 1; sVCAM-1 = soluble vascular cell adhesion protein 1; sVEGFR-1 and -2 = soluble vascular endothelial growth factor receptors 1 and 2; TM = thrombomodulin. Maximum recommended time in months that the 14 analytes can safely be stored (≤ 20% fractional change from baseline) in different conditions based on the results of this study.

* Marker was not stable when assessed at 1 month.

† Recommendation adjusted from 3 to 1 month based on results for IL-8 at other temperatures.

Overall, pairwise comparison of the regression slopes indicated that most biomarkers were less stable when stored in warmer temperatures and in DBS specimens compared with plasma. The concentrations of six of the 13 biomarkers changed faster in plasma stored at –20°C compared with –80°C. Among the 11 biomarkers that could be compared between DBS specimens and plasma, faster rates of change were observed for 8, 9, and 10 of the biomarkers stored in DBS at –20°C, 4°C, and 35°C, respectively (Appendix 4). Although these comparisons are statistically significant, clinically important differences are likely determined by the precision of the assay.

Our results indicate that the 13 host biomarkers tested can be safely stored in plasma aliquots at –20°C for at least 6 months. For 12 of the analytes, longitudinal fluctuations in concentration were within the expected precision of the assay, and for the remaining analyte (sICAM-1), there was no appreciable difference in stability between aliquots stored at –20°C and –80°C. This finding could increase the feasibility of conducting research outside of specialist and tertiary hospitals that lack facilities for –80°C storage. Collection, centrifugation, and storage of plasma at –20°C would be practical at many district-level facilities in LMICs.

In settings where collection and centrifugation of venous blood may be infeasible but access to a refrigerator is possible (e.g., many primary health centers in rural regions of LMICs), DBS specimens may be an attractive option as all biomarkers studied were stable under these conditions for up to one month (Table 1).

Decreasing stability was observed with increasing temperature and storage duration across most analytes when stored as DBS, with two of the analytes (Angpt-1, CRP) demonstrating instability > 20% within 1 month when stored at ambient tropical temperatures. In addition to being faster, rates of change in the concentrations of biomarkers stored as DBS specimens also appeared more variable, which may make application of a “correction factor” challenging (Appendix 5). These findings, which are consistent with previous studies,^10^ illustrate the importance of systematically evaluating analyte-specific field-to-laboratory workflows.

There are several limitations to our work. First, blood samples were obtained from individuals with unknown disease severity and infectious etiologies. As a result, some of the analytes had to be excluded as too few samples had detectable levels at baseline. However, because we evaluated fractional change, we believe that for the analytes studied our findings would be maintained at higher baseline concentrations. Second, although we simulated a range of storage temperatures, we did not consider changes in humidity or the impact of temperature fluctuations—for example, between day and night. Third, although the Luminex platform allowed us to easily quantify multiple biomarkers simultaneously, the interassay variation of the platform limited our ability to detect smaller changes in analyte concentrations. Future work should address these limitations and aim to confirm our findings with a larger sample size, focusing on the markers that have shown greatest promise.

The importance of conducting research at peripheral levels of the health system cannot be overstated. Findings from urban centers where most functional research capacity exists in LMICs should not necessarily be extrapolated to rural settings and populations. Important differences in fever epidemiology and patient characteristics (e.g., nutritional status, vaccination coverage, and care-seeking behavior) mean that it is vital for researchers to collect data directly from their anticipated beneficiaries. The possibility of storing plasma at –20°C and for some analytes using DBS specimens may make such research more feasible.

## Supplemental Material


Supplemental materials
