# Supplementary material for: Practical Methods to Permit the Analysis of Host Biomarkers in Resource-Limited Settings
Source: Am J Trop Med Hyg. 2022 Apr 18;106(6):1765–9. doi: 10.4269/ajtmh.21-1045 (PMC9209911; doi:10.4269/ajtmh.21-1045)
Supplement: Supplementary file 1 [file tpmd211045.SD1.pdf]

Supplemental Appendix 1. Endothelial and immune activation biomarker extraction and quantification protocol from dried blood spots.

Optimization of Extraction Method for Elution of Biomarkers from Dried Blood Spots

As limited literature exists regarding the optimal extraction method for the elution of endothelial and immune activation biomarkers from dried blood spots (DBS), we developed a protocol prior to initiating our stability studies. Multiple extraction buffers, elution times, and DBS extraction buffer volumes were evaluated head-to-head to identify the protocol with optimal analyte recovery using whole blood spiked with analyte of known concentration. For each protocol evaluated, the DBS volume (50 µl) and marker quantification method (Luminex Magpix) remained the same. **Table S1** describes reproducibility and percent recovery for all analytes for the selected protocol. Regardless of the extraction method, soluble intercellular adhesion molecule 1 (sICAM-1), interleukin-10 (IL-10) and chitinase-3-like protein (CHI3L1) could not be fully recovered from the DBS. However, reproducibility of the extraction procedure was excellent as percent coefficient of variation for all analyte replicates ranged from 2.5 to 6.9%.

Supplemental Table S1. Performance characteristics of the dried blood spot extraction protocol selected for the biomarker stability studies.[a]

Analyte	Intra-assay % coefficient of variation	Mean % recovery of analyte[b]
**Angpt-1**	3.6	100.5
**Angpt-2**	2.7	104.2
**CHI3L1**	2.9	72.0
**CRP**	4.7	108.3
**CXCL10**	2.5	99.2
**IL-6**	2.7	103.5
**IL-8**	3.9	108.6
**IL-10**	3.0	89.6
**PCT**	6.9	103.3
**sICAM-1**	4.5	54.4
**sTNFR1**	4.7	110.1
**sTREM-1**	3.3	110.2
**sVCAM-1**	2.9	102.6
**sVEGFR-1**	5.8	98.0
**sVEGFR-2**	3.5	110.8
**TM**	4.7	108.3

[a]Dried blood spots were prepared using 50 µl whole blood and analytes quantified using Luminex Magpix. All experiments were performed in duplicates. [b]Mean % recovery was calculated by spiking whole blood with a known concentration of analyte prior to spotting. Background concentration of analyte in blood was added to the known spiked concentration to obtain the denominator to calculate % recovery.

Biomarker Dried Blood Spot Extraction and Quantification Protocol

Whatman Protein Saver 903 cards were removed from the designated storage temperatures (-20°C, 4°C and 35°C) at each timepoint (one, 3 and 6 months). Two DBS were extracted for each temperature at each timepoint and the mean concentration used for the analysis. Entire 50 µl spots were cut from the protein saver card and transferred to individual wells on a 24-well microtiter plate containing 180 µl extraction buffer (0.01M PBS, 0.5M NaCl, 0.1% v/v Tween 20, pH 7.2). Microtiter plates were placed on a plate shaker (600 rpm) for 2 hours at ambient temperature (22 to 25°C). After incubation, the eluted DBS in the extraction buffer was transferred to 1.5 ml conical tubes and was immediately used to prepare the appropriate dilutions to be quantified by Luminex system (**Table S2**). Quantification of biomarkers by Luminex was performed according to manufacturer instructions.

Supplemental Table S2. Dried blood spot extracts and plasma dilutions used to measure biomarker concentrations using the Magpix Luminex assay.

Analyte	Dilution Factor[a]	
	Plasma	Dried Blood Spot
**Angpt-1**	2	2
**Angpt-2**	2	2
**CHI3L1**	2	2
**CRP[b]**	10,000 or 100,000	200 or 10,000
**CXCL10**	2	2
**IL-6**	2	2
**IL-8**	2	2
**IL-10**	2	2
**PCT**	2	2
**sICAM-1**	2	2
**sTNFR1**	2	2
**sTREM-1**	2	2
**sVCAM-1**	2	2
**sVEGFR-1**	2	2
**sVEGFR-2**	2	2
**TM**	2	2

[a]Plasma and extracted dried blood spots were diluted in R&D Systems Luminex Kit Sample Diluent as described for other specimen types. [b]Due to large variability in peripheral CRP concentrations between samples, two separate dilutions were prepared and evaluated to ensure each specimen was within the assay quantification range.

Supplemental Appendix 2. Flow diagram to illustrate the handling of samples that fell below the lower limit of quantification during the six-month follow-up period. LOD = limit of detection; LLOQ = lower limit of quantification.

Supplemental Appendix 3. Change in concentration for 14 host biomarkers assayed from venous plasma and dried blood spots stored at different temperatures over 6 months. Top row = fitted slopes indicating fractional change from baseline; middle row = absolute fractional change from baseline; bottom row = actual biomarker concentrations. Grey lines indicate change for each individual sample; black lines indicate median with interquartile range indicated by blue shading. Y-axis scales from +60% to -100% chosen for fractional change plots to facilitate comparison with Figure 2 in the main manuscript, however scales for Angpt-2, CRP and IL-8 adjusted due to greater range of fractional change in concentration over follow-up period. DBS = dried blood spot.

Angpt-1

Fractional change from baseline (fitted)

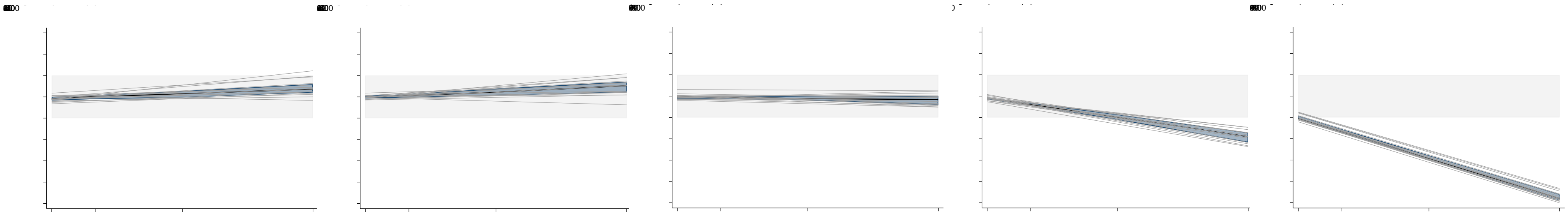

Fractional change from baseline (absolute)

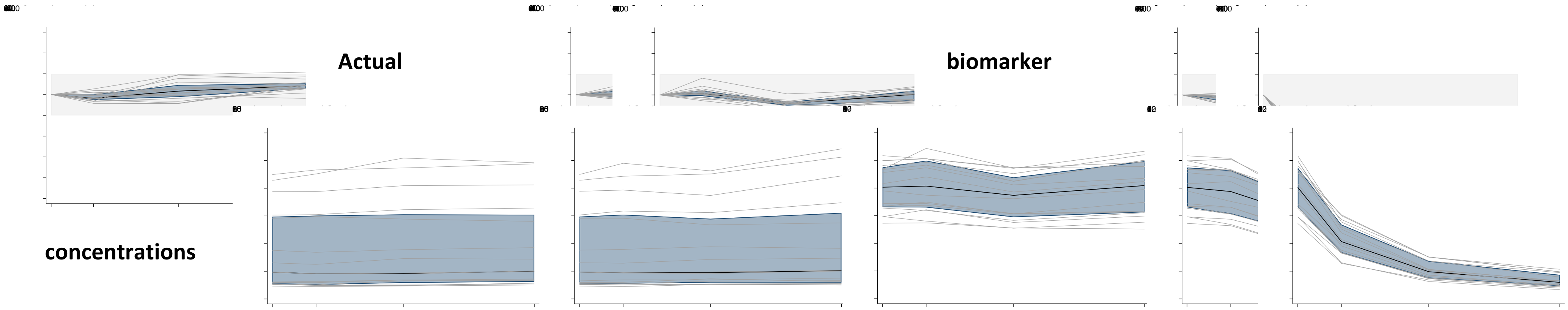

Actual biomarker concentrations

Angpt-2

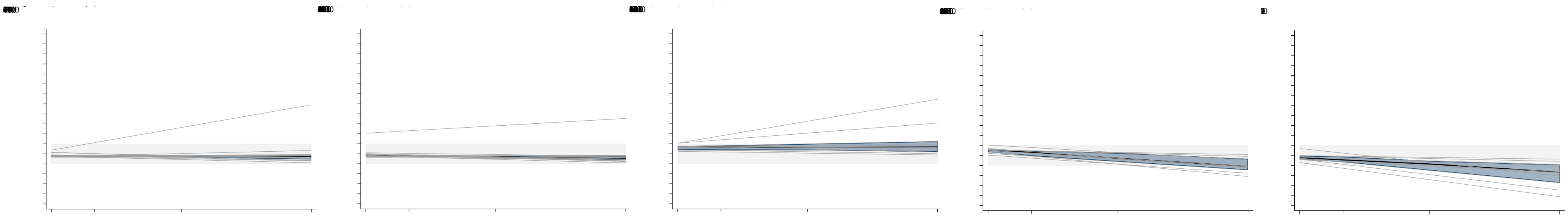

Fractional change from baseline (fitted)

Fractional

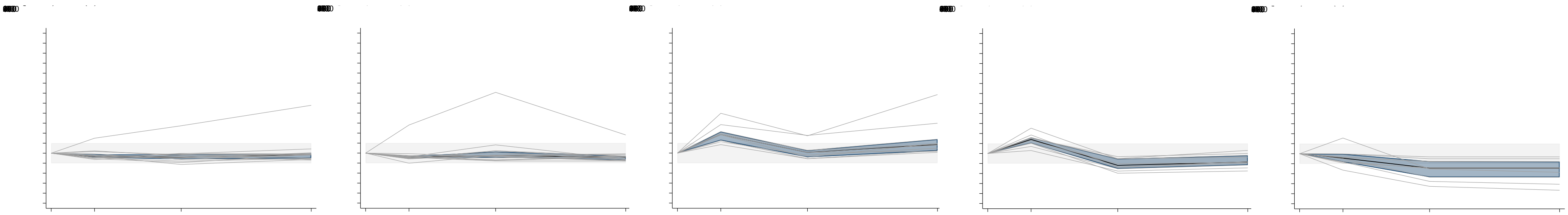

change from baseline (absolute)

Actual biomarker concentrations

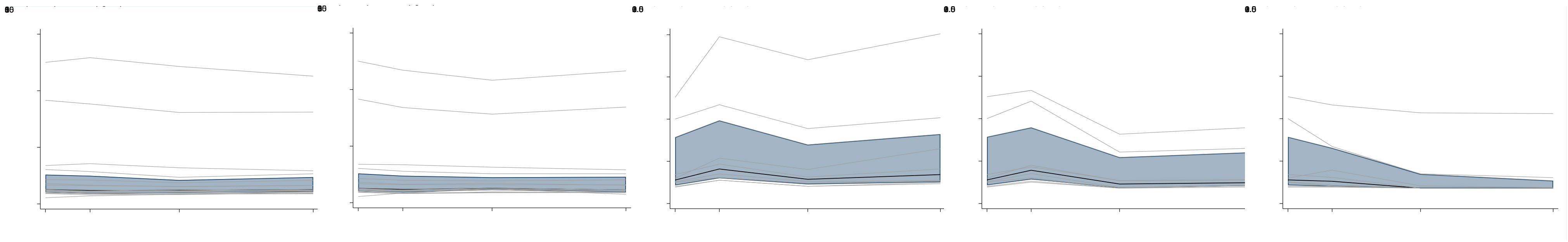

CHI3L1

Fractional change from

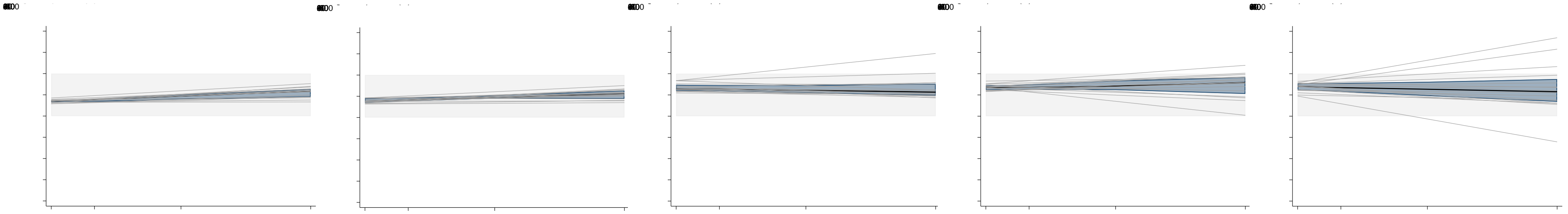

baseline (fitted)

Fractional change from

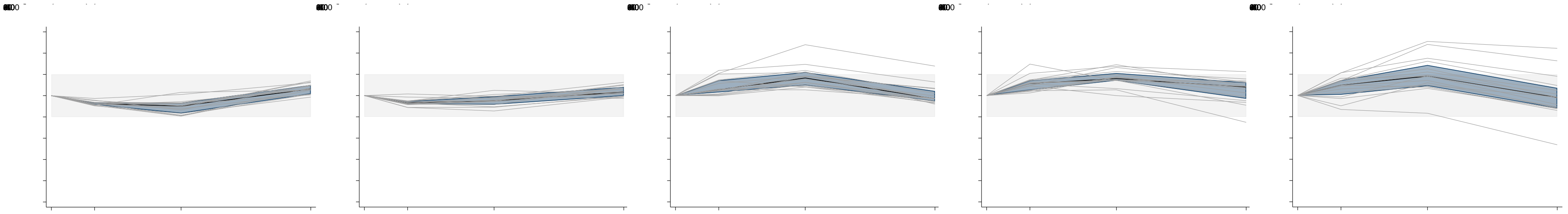

baseline

(absolute)

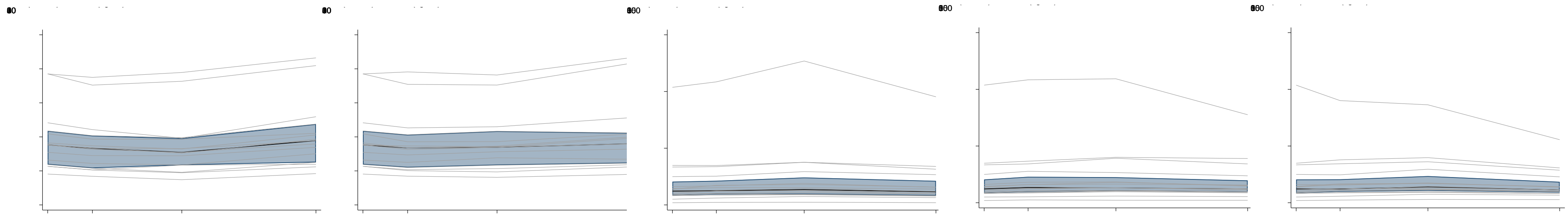

Actual biomarker concentrations

CRP

Fractional change from

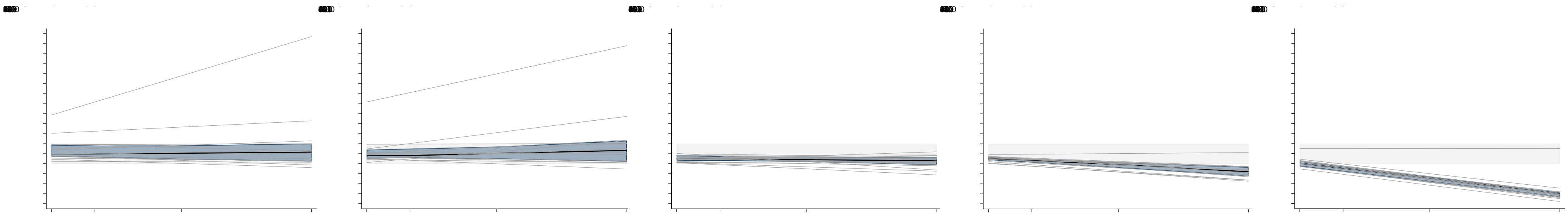

baseline (fitted)

Fractional change from baseline

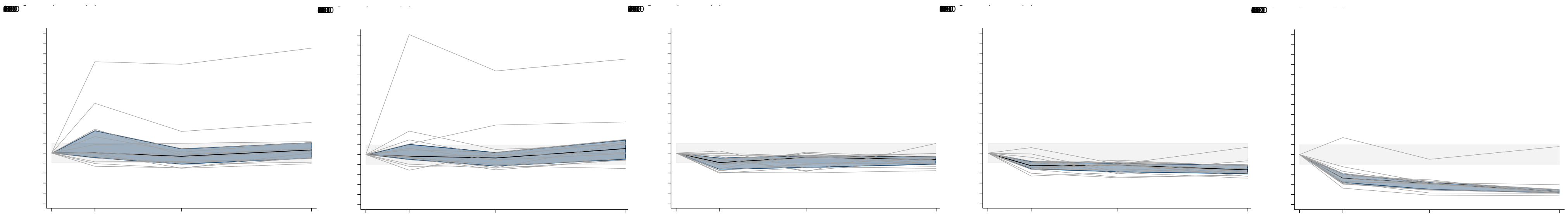

(absolute)

Actual biomarker concentrations

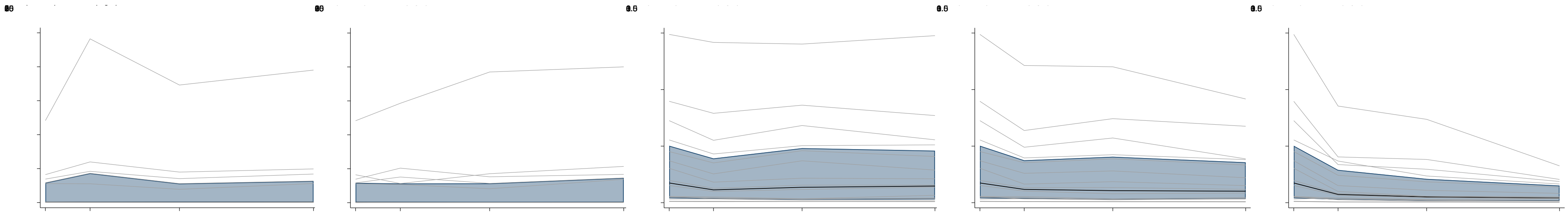

CXCL10

Fractional

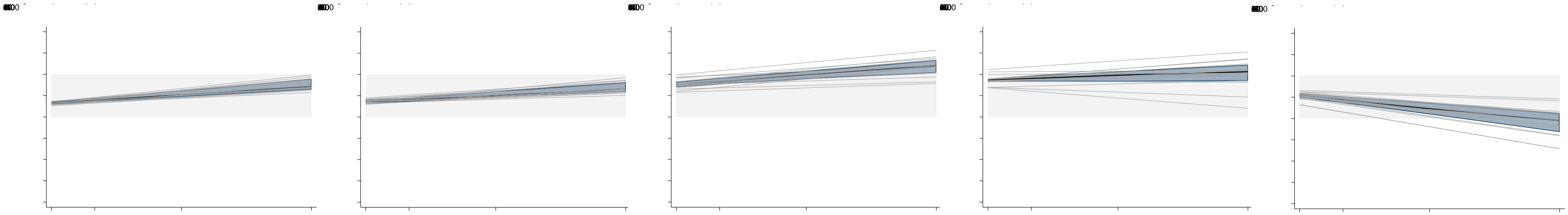

change from baseline (fitted)

Fractional change from baseline (absolute)

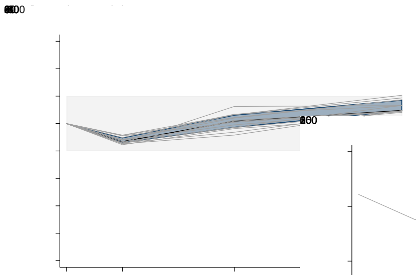

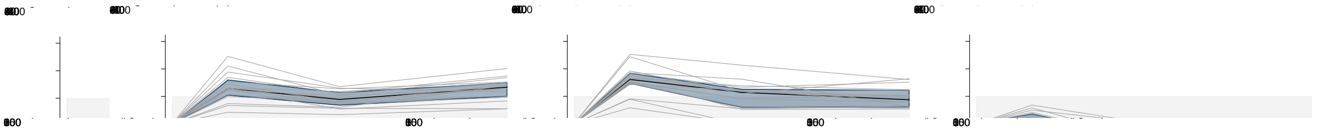

Actual biomarker

concentrations

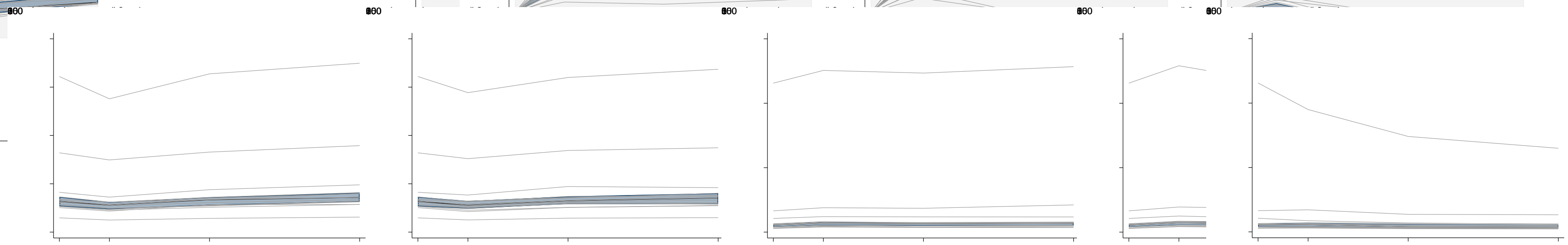

IL-8

Fractional change from baseline (fitted)

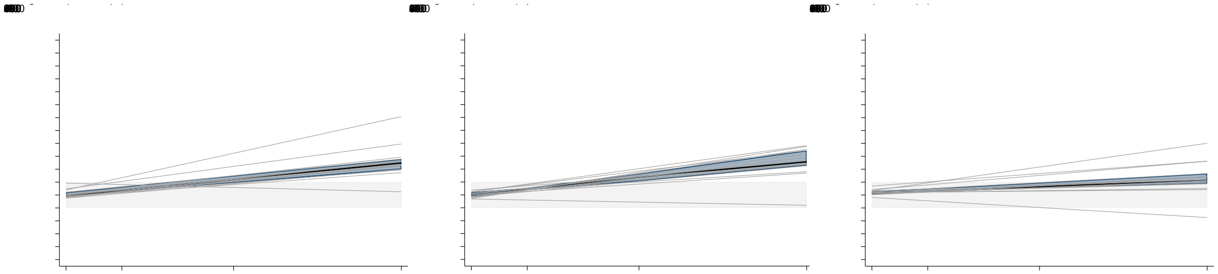

Fractional change from baseline (absolute)

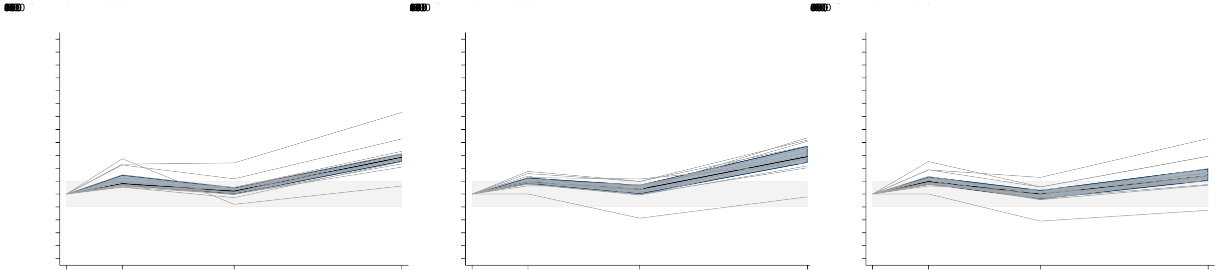

Actual biomarker concentrations

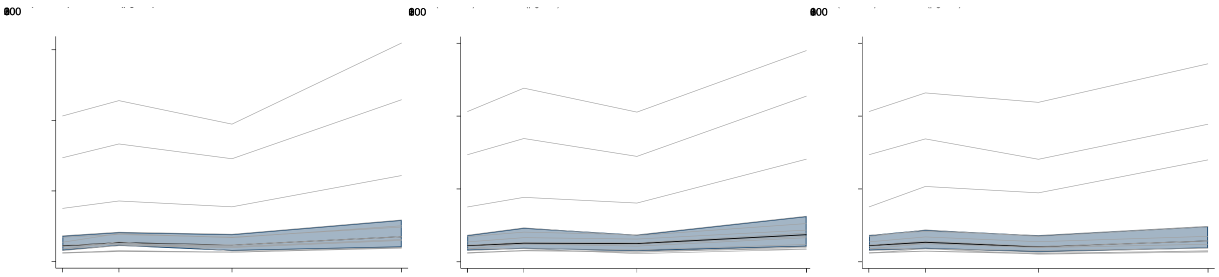

PCT

Fractional change from baseline (fitted)

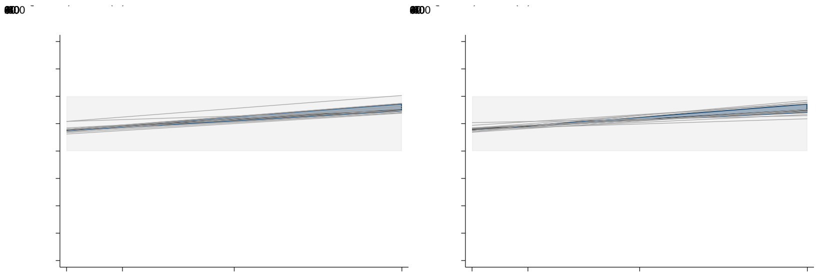

Fractional change from baseline (absolute)

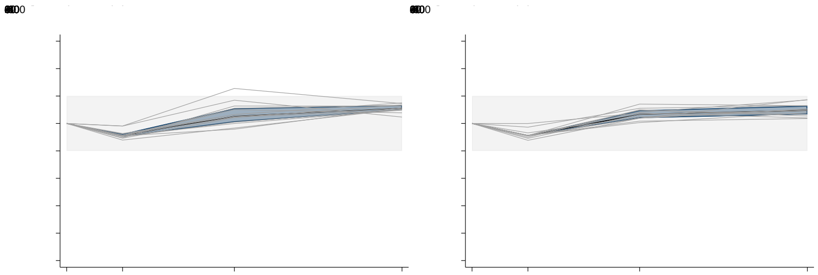

Actual biomarker concentrations

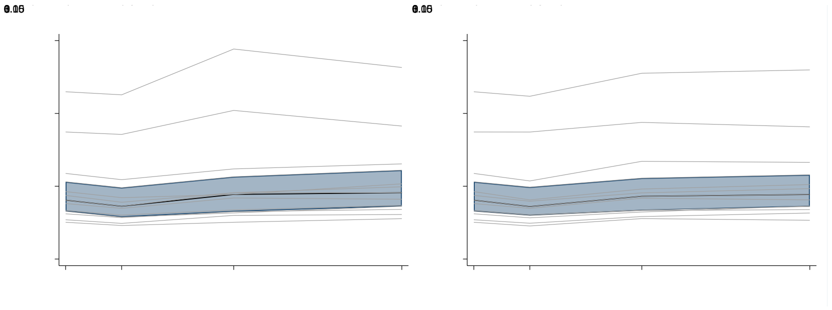

sICAM-1

Fractional change from baseline (fitted)

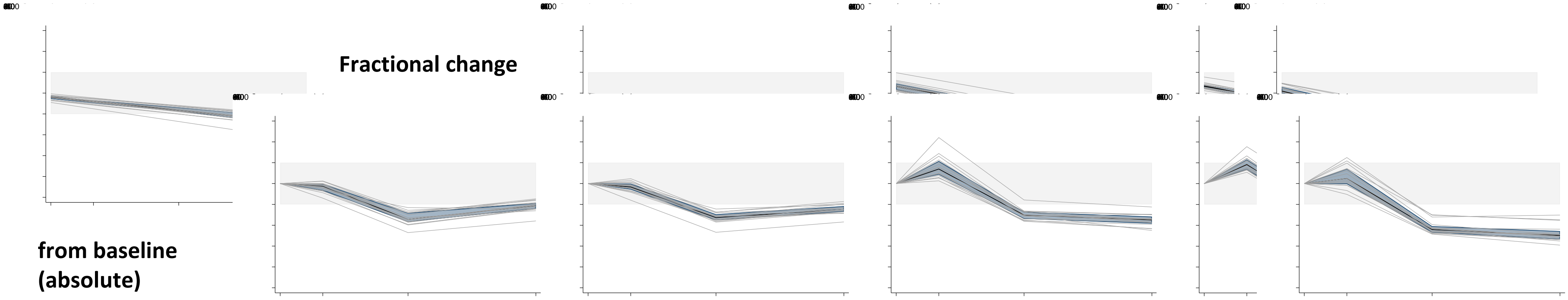

Fractional change

from baseline (absolute)

Actual biomarker concentrations

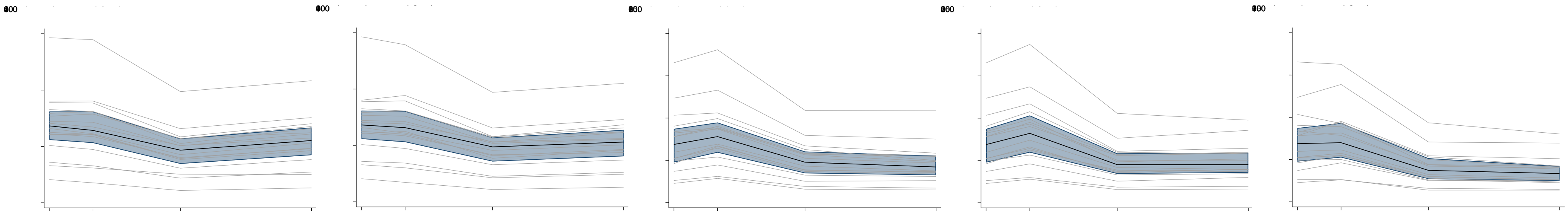

sTNFR1

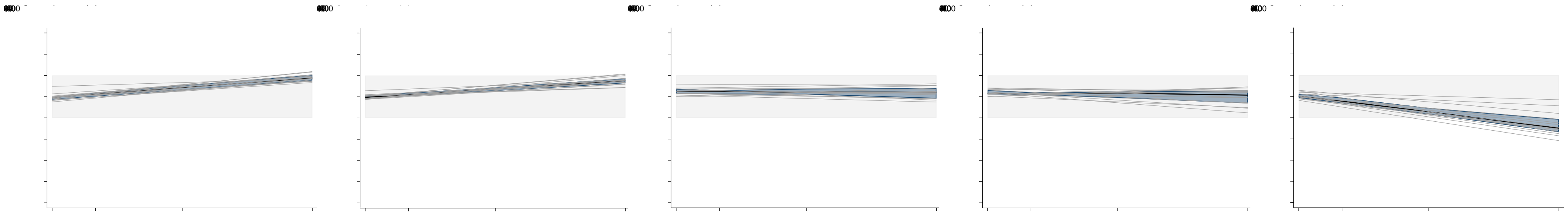

Fractional

change from baseline (fitted)

Fractional change from

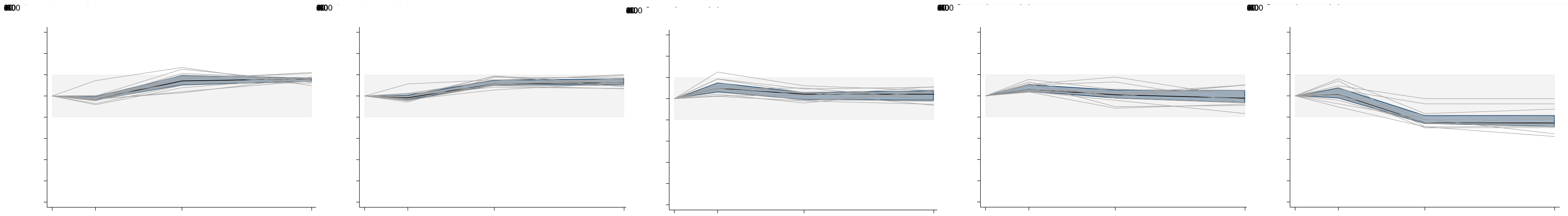

baseline (absolute)

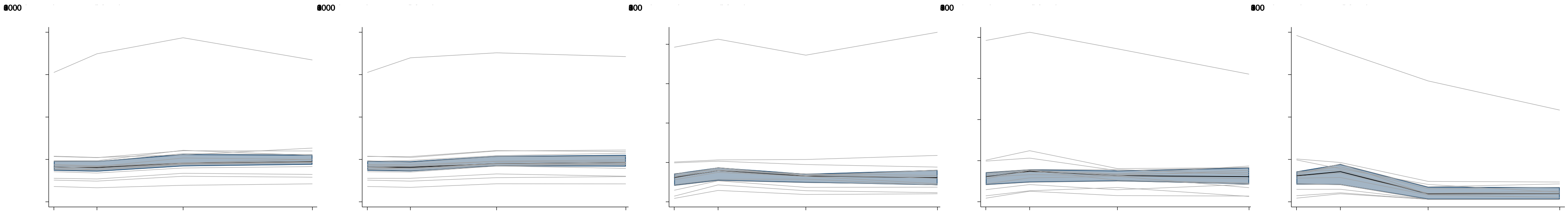

Actual biomarker

concentrations

sTREM-1

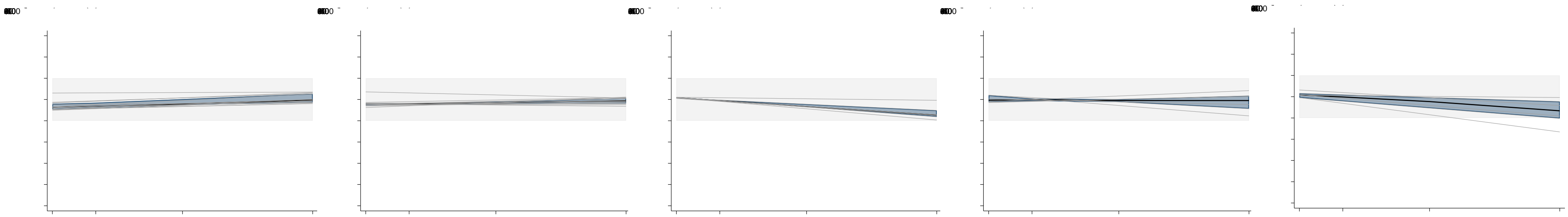

Fractional

change from baseline (fitted)

Fractional change from baseline (absolute)

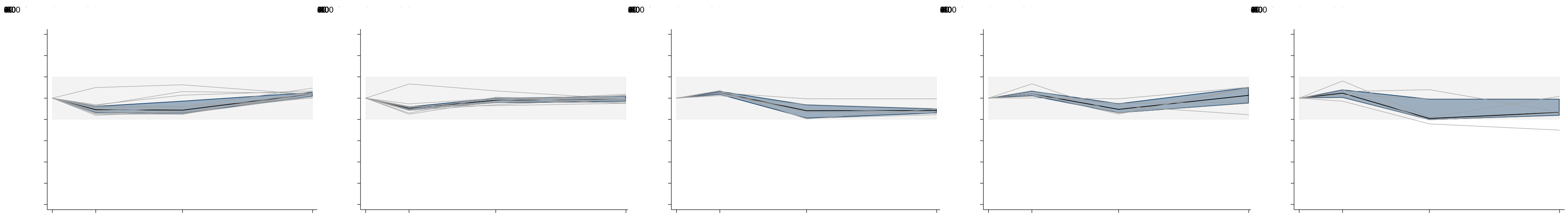

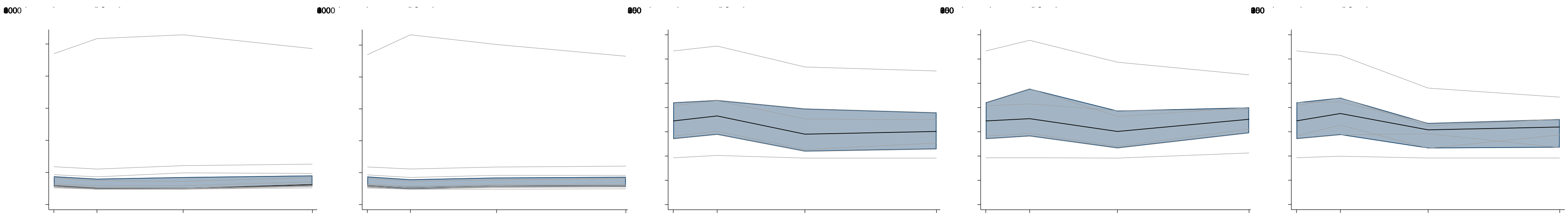

Actual

biomarker concentrations

sVCAM-1

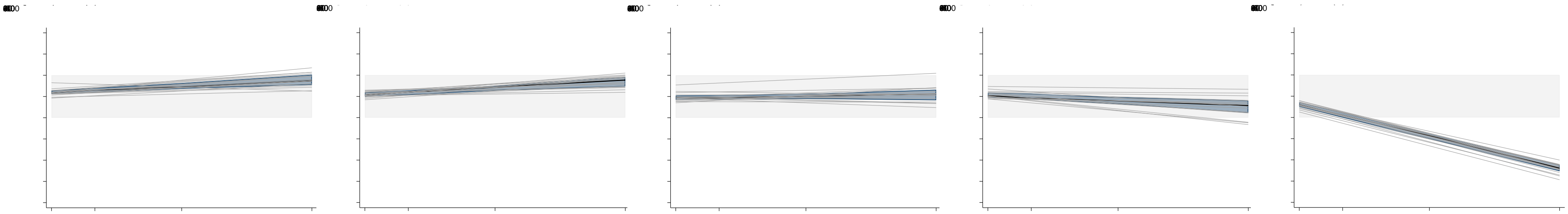

Fractional

change from baseline (fitted)

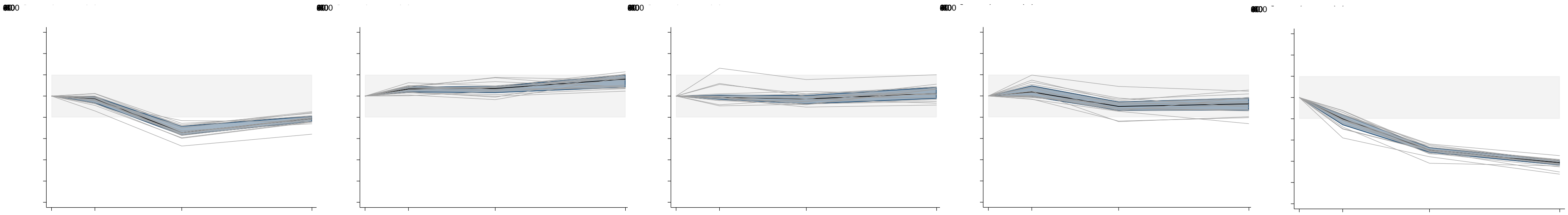

Fractional change from

baseline (absolute)

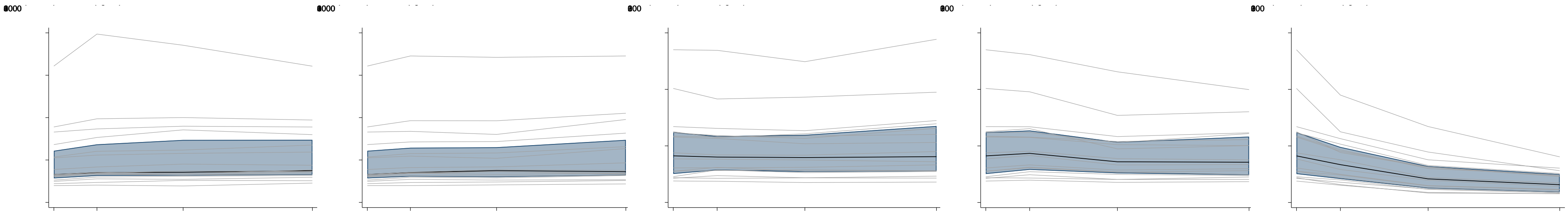

Actual biomarker concentrations

sVEGFR-1

Fractional change from baseline (fitted)

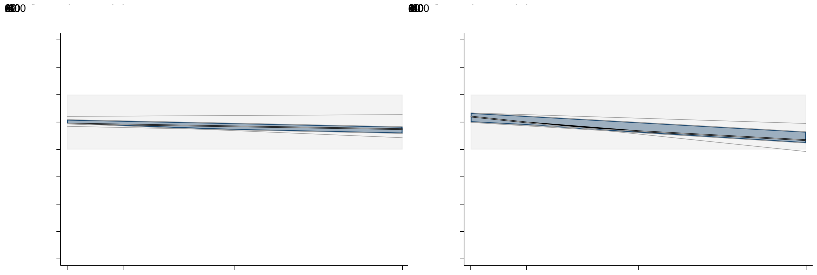

Fractional change from baseline (absolute)

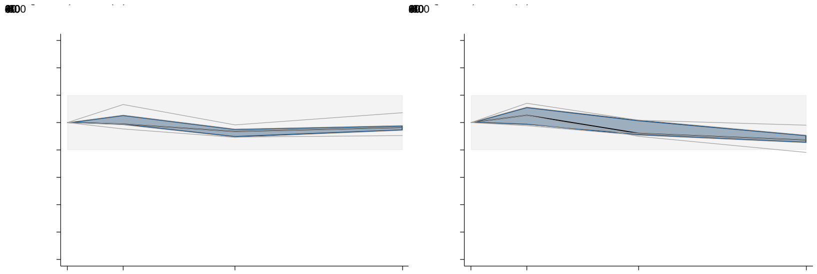

Actual biomarker concentrations

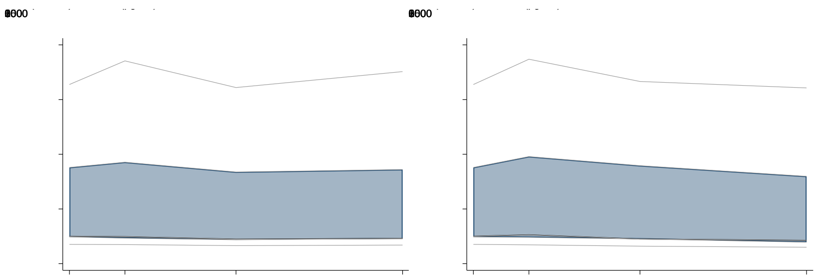

sVEGFR-2

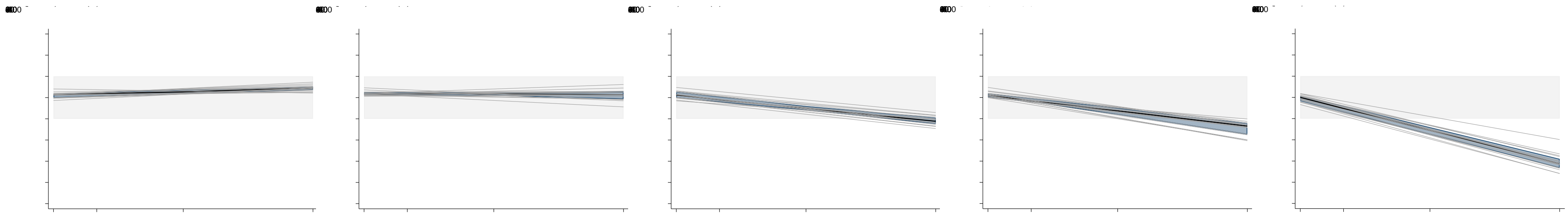

Fractional change

from baseline (fitted)

Fractional change from

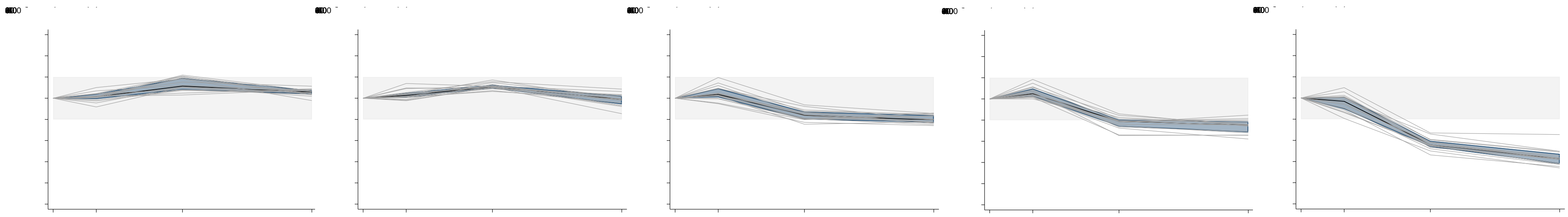

baseline

(absolute)

Actual biomarker

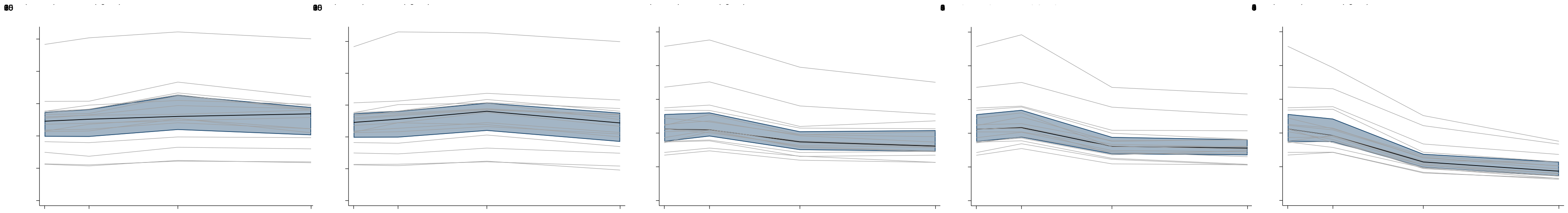

concentrations

TM

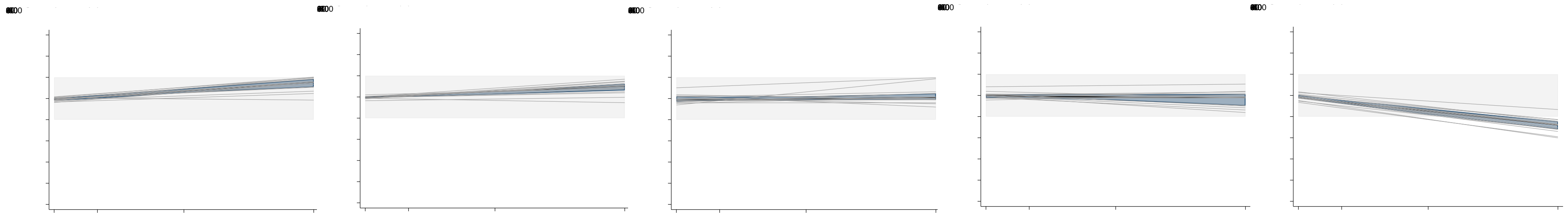

Fractional change from baseline (fitted)

Fractional change from baseline (absolute)

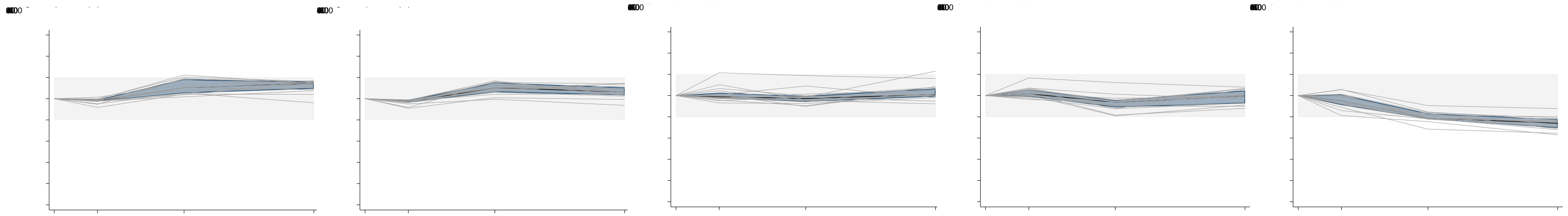

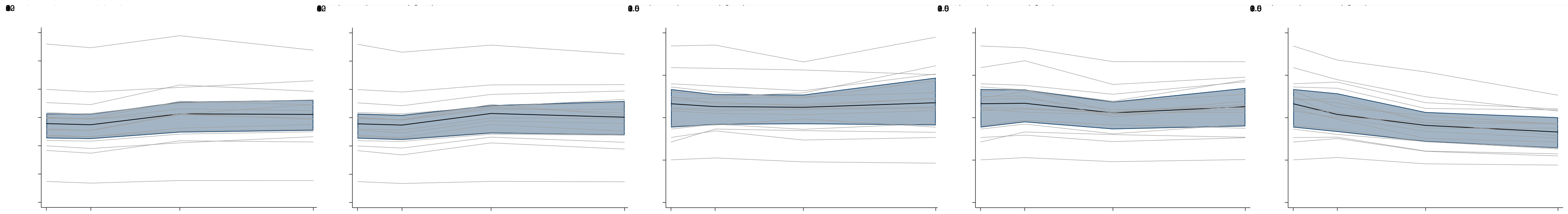

Actual biomarker concentrations

Supplemental Appendix 4. Comparison of median rate of fractional change for biomarkers stored in plasma at -80°C to plasma stored at -20°C and dried blood spot specimens stored at -20°C, 4°C and 35°C. Pairwise comparison performed using Wilcoxon matched-pairs signed-rank test. Plasma stored at -80°C used as reference standard for all comparisons except IL-8, for which DBS -20°C is used. * plasma samples only; ** DBS specimens only. DBS = dried blood spot.

Supplemental Table S3. Comparison of rates of fractional of change.

Biomarker	Plasma -20°C		DBS -20°C		DBS 4°C		DBS 35°C	
	n	p-value	n	p-value	n	p-value	n	p-value
sVEGFR-2	16	**< 0.001**	16	**< 0.001**	16	**< 0.001**	16	**< 0.001**
CHI3L1	15	0.135	15	**0.005**	15	0.107	15	0.073
CXCL10	15	**< 0.001**	15	0.639	15	**< 0.001**	15	**< 0.001**
sVEGFR-1*	5	0.063						
Angpt-2	16	0.058	10	0.064	10	**0.002**	10	**0.002**
PCT*	13	0.340						
TM	16	**< 0.001**	16	**< 0.001**	16	**< 0.001**	16	**< 0.001**
sVCAM-1	16	0.744	16	**0.003**	16	**< 0.001**	16	**< 0.001**
sICAM-1	16	**< 0.001**	16	**< 0.001**	16	**< 0.001**	16	**< 0.001**
CRP	14	0.391	14	0.119	14	**< 0.001**	14	**< 0.001**
Angpt-1	16	0.744	16	**< 0.001**	16	**< 0.001**	16	**< 0.001**
sTREM-1	12	**0.001**	6	**0.031**	6	0.094	6	**0.031**
sTNFR1	16	**0.002**	14	**< 0.001**	14	**< 0.001**	14	**< 0.001**
IL-8**					14	0.715	14	**< 0.001**

Supplemental Appendix 5. Variability of biomarker concentrations over time. Interquartile range of the (fitted) rates of change for each biomarker at one-, three- and six-month timepoints. Note, y-axis scales from 0% to 25% used for all biomarkers, except Angpt-2 and CRP due to greater range of fractional changes in concentration over follow-up period. Interquartile range of rates of change for CRP in plasma larger than for dried blood spots due to outliers. Blue = plasma -80°C; green = plasma -20°C; orange = DBS -20°C; purple = DBS 4°C; red = DBS 35°C. DBS = dried blood spot.

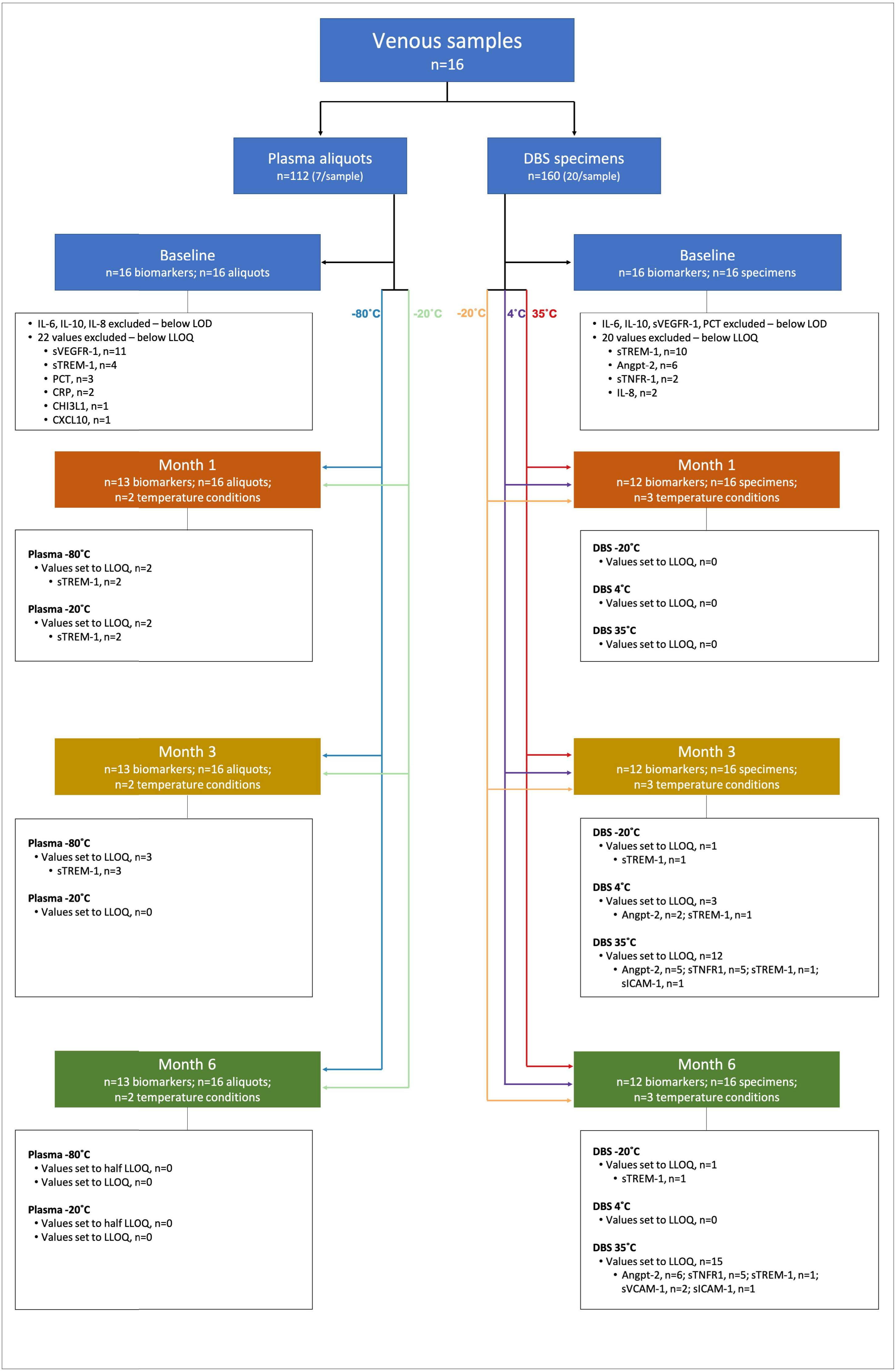

Venous samples
n=16
Plasma aliquots
n=112 (7/sample)
DBS specimens
n=160 (20/sample)
Baseline
n=16 biomarkers; n=16 aliquots
IL-6, IL-10, IL-8 excluded – below LOD
22 values excluded – below LLOQ
sVEGFR-1, n=11
sTREM-1, n=4
PCT, n=3
CRP, n=2
CHI3L1, n=1
CXCL10, n=1
-80˚C
-20˚C
-20˚C
4˚C
35˚C
Baseline
n=16 biomarkers; n=16 specimens
IL-6, IL-10, sVEGFR-1, PCT excluded – below LOD
20 values excluded – below LLOQ
sTREM-1, n=10
Angpt-2, n=6
sTNFR-1, n=2
IL-8, n=2
Month 1
n=13 biomarkers; n=16 aliquots;
n=2 temperature conditions
Plasma -80˚C
Values set to LLOQ, n=2
sTREM-1, n=2
Plasma -20˚C
Values set to LLOQ, n=2
sTREM-1, n=2
Month 1
n=12 biomarkers; n=16 specimens;
n=3 temperature conditions
DBS -20˚C
Values set to LLOQ, n=0
DBS 4˚C
Values set to LLOQ, n=0
DBS 35˚C
Values set to LLOQ, n=0
Month 3
n=13 biomarkers; n=16 aliquots;
n=2 temperature conditions
Plasma -80˚C
Values set to LLOQ, n=3
sTREM-1, n=3
Plasma -20˚C
Values set to LLOQ, n=0
Month 3
n=12 biomarkers; n=16 specimens;
n=3 temperature conditions
DBS -20˚C
Values set to LLOQ, n=1
sTREM-1, n=1
DBS 4˚C
Values set to LLOQ, n=3
Angpt-2, n=2; sTREM-1, n=1
DBS 35˚C
Values set to LLOQ, n=12
Angpt-2, n=5; sTNFR1, n=5; sTREM-1, n=1; sICAM-1, n=1
Month 6
n=13 biomarkers; n=16 aliquots;
n=2 temperature conditions
Plasma -80˚C
Values set to half LLOQ, n=0
Values set to LLOQ, n=0
Plasma -20˚C
Values set to half LLOQ, n=0
Values set to LLOQ, n=0
Month 6
n=12 biomarkers; n=16 specimens;
n=3 temperature conditions
DBS -20˚C
Values set to LLOQ, n=1
sTREM-1, n=1
DBS 4˚C
Values set to LLOQ, n=0
DBS 35˚C
Values set to LLOQ, n=15
Angpt-2, n=6; sTNFR1, n=5; sTREM-1, n=1; sVCAM-1, n=2; sICAM-1, n=1

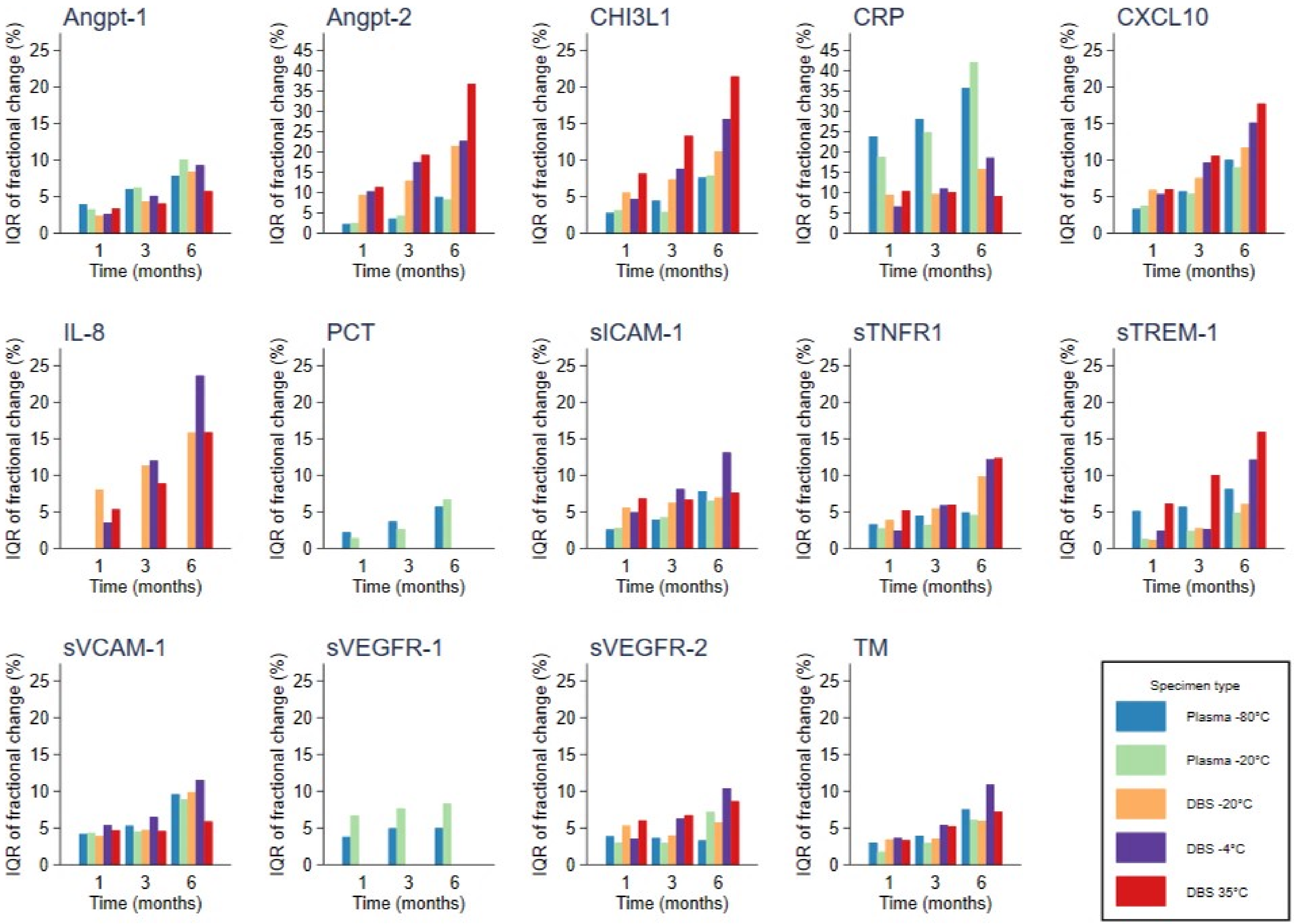

Angpt-1
Angpt-2
CHI3L1
CRP
CXCL10
IL-8
PCT
sICAM-1
sTNFR1
sTREM-1
sVCAM-1
sVEGFR-1
sVEGFR-2
TM
IQR of fractional change (%)
Time (months)
Specimen type
Plasma -80°C
Plasma -20°C
DBS -20°C
DBS -4°C
DBS 35°C
